# Vaccination prevents allergic disorders in children

**DOI:** 10.1186/1939-4551-8-S1-A19

**Published:** 2015-04-08

**Authors:** Olf Herbarth, Adine Marquis, Adine Marquis, Michael Borte, Ulrike Diez, Matthias Richter

**Affiliations:** 1 University Leipzig, Faculty of Medicine, Germany; 2 Public Health Office Berlin-Pankow, Children and Youth Health Service, Germany; 3 Children's Hospital at Municipal Hospital St. Georg Leipzig, Germany; 4 Helmholtzcenter for Environmental Research, Germany

## Background

The association between vaccination and allergic disorders is discussed controversially. That’s why unsurprisingly parents refuse to get their children vaccinated due to the supposed risk of allergies in the later life caused by vaccination. The presented investigation tries to elucidate the potential link between vaccination and allergies.

## Methods

2187 study participants of different birth years (1988/89, 94/95, 98/99) but same age group (5-6 years old) were involved in 3 epidemiological studies using questionnaires, clinical examinations and determination of vaccination titer: a cross sectional study (S1) supplemented by the Leipzig part of a multicenter birth cohort study (S2) and a birth cohort study with children at risk for allergy (S3). Based on questionnaires, vaccination certificates and vaccination titer respectively it was distinguished between no, incomplete and full vaccination. All vaccinations recommended by the German Standing Committee on vaccination have been considered, such as vaccinations against tetanus, haemophilus influenca type B, measles etc. Targets were physician diagnosed atopic eczema and any other allergic symptoms.

## Results

All three studies (S1-S3) showed the lower the vaccination protection the higher the prevalence of allergic diseases. Prevalences (pooled dataset) [%] are for atopic eczema without/with vaccination 29.6 / 22.1 and for allergic symptoms 38.9 / 32.7.

Logistic models have been adjusted for gender, older siblings, passive smoking, smoking during pregnancy, cats, traffic, parental predisposition (apart from S3, parental predisposition was including condition) and such exposures which have shown an association in the past like renovation activities. Vaccination was a significant factor of influence in every study. Considering adjustment the influence of vaccination was on eczema (pooled analysis) adjOR(vacc) [p; 95%CI] 0.66 [0.0013; 0.51…0.85] and on allergic symptoms adjOR(vacc) 0.74 [0.014; 0.59…0.94]. A meta-analysis delivers a PetoOR [95%CI] of 0.71 [0.55…0.92] for eczema and 0.76 [0.60…0.97] for allergic symptoms.

Logistic regression based on vaccination titer shows similar results adjOR 0.69; p=0.002 both for eczema and allergic symptoms.

## Conclusions

All results show that vaccinations do not provoke allergies in later life. On the contrary vaccinations have a protective effect relating to allergies and even in the case of allergic predisposed children.

